# Inherited retinal disease pathway in the UK: a patient perspective and the potential of AI

**DOI:** 10.1136/bjo-2024-327074

**Published:** 2025-05-09

**Authors:** Wendy Wong, Dayyanah Sumodhee, Tiyi Morris, Bhavna Tailor, Catherine Hollyhead, William A Woof, Stephen Archer, Carl Veal, Loy Lobo, Saoud Al-Khuzaei, Malena Daich Varela, Thales A C de Guimaraes, Manuel Gomes, Mital Shah, Mariya Moosajee, Susan M Downes, Savita Madhusudhan, Omar A Mahroo, Andrew R Webster, Michel Michaelides, Nikolas Pontikos

**Affiliations:** 1 University College London Institute of Ophthalmology, London, UK; 2 Genetics, Moorfields Eye Hospital NHS Foundation Trust, London, UK; 3 Department of Ophthalmology, Centre for Innovation, Singapore; 4 Health Economics, UCL, London, England, UK; 5 Eye2Gene Patient Advisory Group, London, UK; 6 Stargardt's Connected, Waltham Cross, UK; 7 University of Oxford, Oxford, UK; 8 Department of Ophthalmology, Faculdade São Leopoldo Mandic, Campinas, SP, Brazil; 9 Liverpool University Hospitals NHS Foundation Trust, Liverpool, UK

**Keywords:** Diagnostic tests/Investigation, Surveys and Questionnaires, Genetics

## Abstract

**Background:**

Inherited retinal diseases (IRDs) are the leading cause of blindness in young people in the UK. Despite significant improvements in genomics medicine, the diagnosis of these conditions remains challenging, and around 40% do not receive a definite genetic diagnosis after extensive genetic testing. This survey aims to investigate the experience of individuals affected by IRDs, their relatives, friends and caregivers, focusing on their care and diagnostic journey. Additionally, it explores the potential acceptability of artificial intelligence (AI) technologies, such as Eye2Gene, that predict causative genes from retinal images of patients with IRDs.

**Methods:**

This cross-sectional survey included Likert scale and open-ended questions and was distributed electronically using the Qualtrics platform between April and August 2024. The survey included questions on respondent demographics; their journey to receive specialist care and genetic testing; their information needs and their attitude towards AI-augmented diagnosis. Descriptive statistics and content analysis were used to interpret the survey responses.

**Results:**

The survey had 247 responses, of which 242 were analysed after removing four duplicates and one without consent; 80.2% were patients and the remainder were relatives, friends or caregivers. There was substantial variability in patient diagnostic journeys in terms of waiting times to see a specialist (IQR, 1–4 years), commute required (IQR, 10–74 miles) and number of visits to reach a diagnosis (IQR, 2–4). A substantial proportion of patients (35.8%) had a change in diagnosis. The majority of respondents (>90%) were overwhelmingly in favour of the integration of AI into the IRD pathway to accelerate genetic diagnosis and improve care.

**Conclusion:**

This survey identifies several key gaps and disparities in the IRD care pathway which may potentially be bridged with AI. The survey also reveals a favourable attitude towards incorporating AI into diagnostic testing of IRDs.

**WHAT IS ALREADY KNOWN ON THIS TOPIC**

- Inherited retinal diseases are a leading cause of blindness in the working age population and children in the UK. They are now routinely diagnosed in the National Health Service thanks to the availability of genetic testing.

**WHAT THIS STUDY ADDS**

- This study found that, despite significant recent improvements in genomics medicine, large disparities remain in the diagnostic pathway in terms of the diagnostic journey, the communication of genetic testing and results. The prospect of an imaging-based AI-augmented diagnosis pathway for inherited retinal disease is well received.

**HOW THIS STUDY MIGHT AFFECT RESEARCH, PRACTICE OR POLICY**

- The findings of this study inform the need to standardise the inherited retinal disease pathway in the UK and highlight the potential role of artificial intelligence in achieving this.

## Introduction

 Inherited Retinal Diseases (IRDs) are a significant cause of irreversible vision loss, affecting up to one in 1380 individuals worldwide and representing a leading cause of blindness in the working-age population and children.^1^,^4^ The term IRD encompasses a large group of genetically predetermined retinal conditions that have been linked to pathogenic variants in over 300 different genes.^5 6^ The International Rare Diseases Research Consortium has set a commendable target of reducing the diagnostic delay for known medical disorders to under 1 year, but the average time to diagnosis for an IRD patient in Europe or America is presently 6.4±9.1 years.^7 8^ Reaching a definitive diagnosis for patients with IRDs within the recommended 1-year timeframe is challenging because of extreme genetic heterogeneity, pronounced phenotypic variability and limited available clinical expertise for IRDs.^9^

Although next-generation sequencing technology offers a powerful tool to shorten the diagnostic process for patients with IRD, a genetic diagnosis is still not obtained in 26–48% of individuals globally.^10^ Also, the processes used to analyse the sequencing data can significantly influence the diagnostic yield.^11^ Another challenge lies in interpreting novel or rare genetic variants, which can yield an inconclusive result as a variant of uncertain significance. Some of this ambiguity can be resolved where the patient’s phenotype is highly specific for the disease and can be used to support the pathogenicity of the detected variants.^12^ The importance of evaluating the phenotype in variant interpretation is also evident from the guidelines jointly published by the American College of Medical Genetics and Genomics and the Association for Molecular Pathology, stating that ‘clinical laboratories are encouraged to form collaborations with clinicians to provide clinical information to better understand how genotype influences clinical phenotype and resolve differences in variant interpretation’.^12^

Multimodal imaging techniques are an important tool in obtaining phenotypic information and valuable diagnostic markers when diagnosing IRDs.^13^ The significant processing power and speed of currently available artificial intelligence (AI) based techniques can analyse large volumes of multimodal imaging data acquired from patients with IRDs, enabling identification of distinct patterns of neuroretinal degeneration and genotype-phenotype correlations.^14 15^ These developments in AI technology can potentially aid in accelerating the diagnostic process while also providing more equitable access to diagnostic expertise for patients with IRDs. Moreover, AI analysis can potentially help in interpreting genetic results to obtain a molecularly confirmed diagnosis in the currently ‘unsolved cases’. This is of significant importance as obtaining a genetic diagnosis helps provide information on the inheritance pattern, supports family planning decisions, enables clinicians to provide prognostic information, facilitates enrolment to disease registries and is required for recruitment to clinical trials.^16^ One such AI tool that is in development to aid genetic diagnosis is Eye2Gene; this tool aims to enhance the diagnostic process by using imaging features to suggest possible genetic diagnosis and support molecular testing. Therefore, the purpose of this study is to identify gaps in the existing IRD care pathway and evaluate patients’ perspectives on using AI to bridge these gaps.

## Methods

### Survey design

The survey was designed and iteratively refined through a three-stage process.^17^ The first stage involved an expert working group arising from the collaboration between a bioinformatician specialised in artificial intelligence (NP), a health psychologist (DS) and ophthalmologists, specialising in IRDs (ARW, MM and OAM). The second stage included patient advisory groups (BT, CH, SAK, CV and LL) to obtain feedback on the clarity of the questions, to ensure the survey adequately captured the patient perspective and that all questions were framed with appropriate sensitivity. The final stage was a pilot to identify any areas of improvement and to obtain an estimate of the time required to complete the survey.

The survey was designed on Qualtrics (Provo, Utah, USA), which dynamically presents the next question based on the submitted response. This adaptive approach was chosen to reduce the burden on respondents by only showing relevant questions and to increase the likelihood of the survey participants completing the survey in its entirety. The survey included a mix of closed and open-ended questions. A Likert scale was used for opinion-related questions. There was a total of 8 main questions, each with multiple subparts (online supplemental information).

### Survey distribution

The survey was distributed electronically using an online secure platform (Qualtrics), through the email distribution lists of registered charities in the UK which provide support to patients with IRDs: Retina UK, Moorfields Eye Charity and Stargardt Connected, to include individuals affected by IRDs, their relatives, friends and caregivers.

### Data analysis

Data were downloaded from Qualtrics, exported into a Microsoft Excel file and examined for duplicate responses by checking if the internet protocol address indicated multiple submissions from the same survey respondent. After excluding duplicate responses, quantitative responses were analysed using pivot tables in Excel.

## Results

Between 22 April 2024 and 12 Aug 2024, 247 fully completed responses were received. The survey was answered by 242 respondents (after removing five submissions). Five responses were excluded from the analysis: four were duplicates and one respondent did not provide consent to participate.

### Demographic characteristics of the survey respondents

The majority of respondents (80.2%) were patients, the remaining were relatives, caregivers or close friends (table 1). The median (IQR) age of the participants was 53 (36–64) years and 57.4% were women. The most common conditions were Stargardt disease (44.2%) and retinitis pigmentosa (RP) (43.0%).

**Table 1:** Demographics and characteristics of survey respondents

Demographics and characteristics	
Gender	**Number (%**)
Female	139 (57.4)
Male	103 (42.6)
Ethnicity	**Number (%**)
White	210 (86.8)
Asian or Asian British	19 (7.9)
Black, black British, Caribbean or African	4 (1.7)
Mixed or multiple ethnic groups	4 (1.7)
Other	4 (1.7)
Prefer not to say	1 (0.4)
Age, years	
Mean (SD)	48.5 (19.8)
Median	53
IQR	36–64
Background of respondent	**Number (%**)
Patient	194 (80.2)
Relative	32 (13.2)
Caregiver	14 (5.8)
Close friend	2 (0.8)
Submitted clinical diagnosis	**Number (%**)
Stargardt disease	107 (44.2)
Retinitis pigmentosa	104 (43.0)
Other	24 (9.9)
Not sure	4 (1.7)
Achromatopsia	2 (0.8)
Leber’s congenital amaurosis	1 (0.4)

SD, Standard Deviation.

### Diagnostic journey

There was significant variability in the number of consultations required to reach a diagnosis, ranging from 1 to 50, with a median of 3 (IQR: 2–4). It was observed that two respondents had indicated achromatopsia as the underlying IRD, and both reported that more than 10 consultations were required to reach a diagnosis. Similarly, the number of years to be seen by a specialist and the distance travelled to see a specialist varied widely, with a median of 1 year (IQR: 1–4) and 25 miles (IQR: 10 to 75), respectively (table 2).

**Table 2:** Diagnostic experience

Diagnostic experience	
Number of consultations for diagnosis	
Mean (SD)	3.7 (5.9)
Median	3
IQR	2–4
Number of years to see a specialist	
Mean (SD)	5.2 (11.0)
Median	1
IQR	one to 4
Travelling distance to see specialist, miles	
Mean (SD)	73.9 (123.1)
Median	25
IQR	ten to 74
Received a change in diagnosis	
Yes	88 (35.8%)
No	158 (64.2%)
Offered a genetic test	
Yes	
Proceeded with test	186 (75.6%)
Did not proceed with test	5 (2.0%)
No	55 (22.4%)
Genetic counselling before testing	
Yes	103 (41.9%)
No	83 (33.7%)
Nil response	60 (24.4%)
Year of genetic testing	
1980 to 1999	15 (6.1%)
2000 to 2009	19 (7.7%)
2010 to 2019	88 (35.8%)
2020 to 2024	68 (27.6%)
Not applicable	61 (24.8%)
Age at receiving results of genetic test	
Mean (SD)	47.5 (19.2)
Median	50
IQR	38 to 58

IQR, Interquartile Range; SD, Standard Deviation.

The diagnosis was revised in over a third of patients (35.8%). Not all patients were offered genetic testing (22.4%), but among those who were offered testing (77.6%), only a small percentage declined (2.6%, 5 out of 191). The majority who underwent genetic testing (72.4%) had the test performed between 2010 and 2020. The median age was 42 years (IQR: 21–54) at the time they received the results of the genetic testing. The two most frequent diagnoses were Stargardt disease and RP, for which the median age at diagnosis was 31 years (IQR: 15–48) and 50 years (IQR: 38–58), respectively.

### Communication of genetic testing and results

One-third of respondents did not undergo or recollect undergoing counselling prior to genetic testing (table 3). Genetic test results were most frequently communicated verbally (in person, over the phone or via teleconference) in 39.9% of cases, followed by written correspondence (letter or electronic mail) in 27.3%. Where the results were communicated during an appointment, the median length of time was 20 min (IQR: 0–30 min).

**Table 3:** Patient communication and information needs

Patient communication and information needs	
Genetic counselling before testing	**Number (%**)
Yes	103 (56.0%)
No	81 (44.0%)
Communication of test results	**Number (%**)
In person	70 (38.0%)
Letter	62 (33.7%)
Over the phone	18 (9.8%)
Via teleconference	9 (4.9%)
Email	8 (4.3%)
Results not received	5 (3.3%)
Do not remember	4 (2.2%)
No response	8 (4.3%)
Length of appointment for communication of results (minutes)	
Mean (SD)	23.1 (24.4)
Median	20
IQR	0 to 30
Perception on understanding of results Number (%)	
Definitely yes	81 (33.5%)
Probably yes	47 (19.4%)
Might or might not	16 (6.6%)
Probably not	23 (9.5%)
Definitely not	17 (6.7%)
Not applicable	58
Perception if questions were adequately answered Number (%)	
Definitely yes	56 (23.1%)
Probably yes	52 (21.5%)
Might or might not	39 (16.1%)
Probably not	20 (8.3%)
Definitely not	17 (7.0%)
Not applicable	58
Preferences on being informed of progression quantitatively not qualitatively	
Prefer a great deal	112 (46.3%)
Prefer a lot	54 (22.3%)
Prefer a moderate amount	42 (17.4%)
Prefer slightly	22 (9.1%)
Do not prefer	12 (5.0%)

IQR, Interquartile Range; SD, Standard Deviation.

About half of the respondents indicated that they probably or definitely understood the results (52.8%), and a slightly smaller proportion indicated that they definitely or probably felt their questions had been adequately answered (44.3%).

A content analysis of respondents’ replies regarding information they seek during follow-up visits revealed these to be the most frequently mentioned topics: treatment (35 responses); research (30 responses) or trials (20 responses); progression or deterioration (29 responses) or prognosis (16 responses) and family (11 responses).

### Attitude towards imaging-based and AI-augmented diagnosis

The vast majority of respondents indicated that they find it acceptable (80.6%) or somewhat acceptable (14.0%) to use ocular imaging to assess the likelihood of an IRD. Only a small percentage (1.6%) indicated they would find this unacceptable or somewhat unacceptable (table 4). With regards to the patients willing to obtain their imaging scans, a majority (65.3%) were in favour of receiving them. Most respondents (78.9%) were supportive of having retinal scans acquired locally sent to the hospital, but 11.2% were undecided and 9.9% expressed some opposition.

**Table 4:** Patient perception on imaging and incorporation of artificial intelligence

Patient perception	
Preliminary assessment of IRD status using an eye scan Number (%)	
Acceptable	195 (80.6%)
Somewhat acceptable	34 (14.0%)
Neither unacceptable nor acceptable	9 (3.7%)
Somewhat unacceptable	2 (0.8%)
Unacceptable	2 (0.8%)
Perceived benefit of Eye2Gene artificial intelligence for diagnosis Number (%)	
Strongly agree	171 (70.7%)
Somewhat agree	47 (19.4%)
Neither agree nor disagree	19 (7.9%)
Somewhat disagree	1 (0.4%)
Strongly disagree	4 (1.7%)
Preference on access to undergoing retinal scans Number (%)	
Definitely yes	158 (65.3%)
Probably yes	57 (23.6%)
Might or might not	18 (7.4%)
Probably not	7 (2.9%)
Definitely not	2 (0.8%)
Preference for local retinal scans to be sent to hospital Number (%)	
Definitely yes	124 (51.2%)
Probably yes	67 (27.7%)
Might or might not	27 (11.2%)
Probably not	15 (6.2%)
Definitely not	9 (3.7%)
Perceived acceptability of doctors using Eye2Gene Number (%)	
Acceptable	179 (74.0%)
Somewhat acceptable	45 (18.6%)
Neither unacceptable nor acceptable	11 (4.5%)
Unacceptable	4 (1.7%)
Somewhat unacceptable	3 (1.2%)

AI, Artificial Intelligence; IQR, Interquartile Range; IRD, Inherited Retinal Disease; RP, Retinitis Pigmentosa; SD, Standard Deviation.

A majority of respondents strongly agreed (70.7%) or somewhat agreed (19.4%) that incorporating AI would be beneficial if it can help clinicians reach the right diagnosis earlier; only a small proportion of respondents (2.1%) expressed disagreement. Most respondents deemed the use of Eye2Gene as acceptable (74%) or somewhat acceptable (18.6%), and only a small minority found it unacceptable (2.9%).

## Discussion

The IRD pathway in the UK is highly variable, meaning that patients can experience significantly different diagnostic journeys depending on the healthcare provisions available locally to them. These differences include the waiting time to see a specialist, the commute involved to see a specialist, and the number of visits needed to obtain a diagnosis. Furthermore, a substantial proportion of patients were initially given a diagnosis that was later changed. These findings are consistent with the previous literature investigating the diagnostic process of patients with IRDs.^8 18^ Incorporating AI techniques, such as Eye2Gene (an AI tool currently under development for IRDs), that are capable of assessing retinal images for features of IRD can help streamline the diagnostic process, support clinical decision-making and improve patient access to expertise in IRD and thus standardise healthcare delivery.^15^

Survey respondents demonstrated a positive attitude towards AI, with a clear majority receptive to its incorporation into the diagnostic testing of IRDs, especially if it assists the clinician in reaching the correct diagnosis quicker. Although most patients are receptive to data sharing and AI in their healthcare, some have reservations. Although this survey did not specifically examine their reasons, it highlights the need to obtain informed consent instead of assuming patient agreement. These results are useful for several stakeholders, including healthcare providers, AI developers and regulatory bodies, as they inform the planning of the implementation of AI in the care of patients with IRDs. Although patients are generally receptive about AI in healthcare, it is imperative that clinicians are trained to implement it responsibly.

This survey identified key topics of interest to patients during consultations, including clinical trials and research developments. This topic on treatment possibility encompasses a vast, growing body of knowledge because of the genetic heterogeneity of IRDs and the rapidly increasing number of clinical trials, making it challenging for clinicians to stay fully informed.^19^ Supporting this point, the study revealed only 44.3% of patients were satisfied that their questions had been adequately addressed. Progress in AI and natural language processing may provide the means to achieving personalised, evidence-based medicine in IRD clinics by efficiently informing clinicians of a real-time summary of the literature relevant to the particular genetic diagnosis.^20 21^ These findings provide invaluable insight for developing and improving AI tools intended for clinicians managing IRDs or patients affected by these conditions.

This survey additionally found that 68.29% of respondents are not only keen to understand the disease trajectory but would also often prefer quantitative measures of progression instead of vague descriptions of deterioration (table 3). While disease progression can be quantified objectively by measuring retinal pigment epithelial atrophy expansion or ellipsoid zone loss, these manually labourious and time-consuming processes are often not possible in busy clinic settings.^22^ However, novel AI approaches may make it possible for clinicians to efficiently chart changes in these key disease features from clinical investigations, allowing for quantitative monitoring of disease progression.^23^ Furthermore, the advent of wearable devices which collect detailed information on the visual behaviour of the patient could greatly enrich and complement the structural insights provided by ocular imaging, offering a more comprehensive basis for disease prognostication.^24^

To our knowledge, this survey is the first to evaluate patient perspectives and considerations regarding the application of AI in the diagnosis of IRDs. This survey includes a relatively large sample size for a rare disease study. Nonetheless there are several limitations to this study. First, the survey was only distributed electronically. This may introduce a sampling bias which favours individuals who are more inclined to adopt technological advances. Second, as this survey was distributed through UK-based charities, these findings may not be generalisable to other healthcare systems and regions outside the UK. Finally, recall bias may be present, as a substantial proportion of patients underwent genetic testing before 2020.

In conclusion, this study demonstrates substantial variations in the time it takes to obtain a diagnosis and the way that information is delivered and understood by patients evaluated in this study. AI algorithms, such as Eye2Gene, have the potential to address these unmet needs and potentially standardise the pathway to offer more patients equitable management and care. The majority of the patients, as well as their companions, have a positive attitude towards AI being incorporated to improve their clinical care in the UK. These findings, which reflect the perspectives of patients, are valuable for informing and shaping policy on the integration of AI into the IRD care pathway.

## Supplementary material

10.1136/bjo-2024-327074 online supplemental file 1

## Data Availability

All data relevant to the study are included in the article or uploaded as supplementary information.
