# Supplementary material for: Inherited retinal disease pathway in the UK: a patient perspective and the potential of AI
Source: Br J Ophthalmol. 2025 May 9;109(11):e327074. doi: 10.1136/bjo-2024-327074 (PMC12573410; doi:10.1136/bjo-2024-327074)
Supplement: online supplemental file 1 [file bjo-109-11-s001.pdf]

Survey Summary Report

Note that not all questions/answers have been included to protect the privacy of the respondents.

Q4.2 - What is [Field-pronoun] inherited retinal condition?

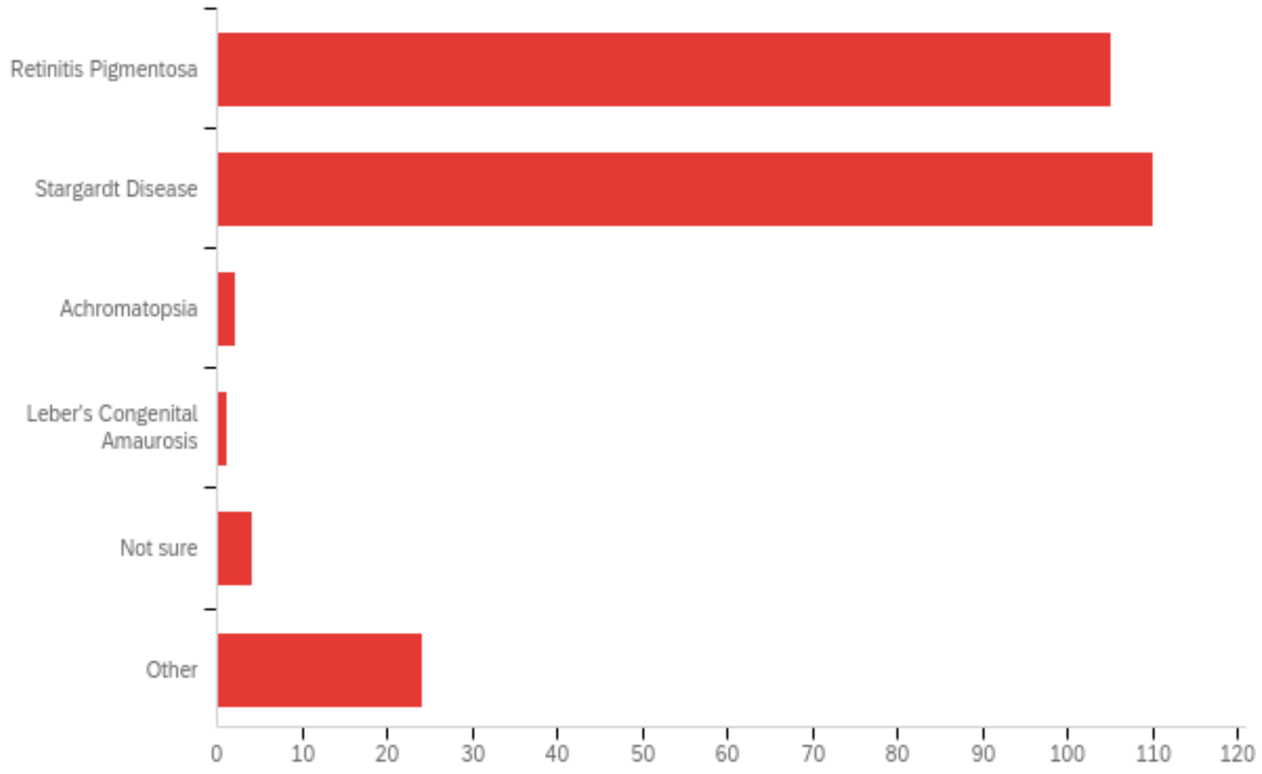

#	Field	Minimum	Maximum	Mean	Std Deviation	Variance	Count
1	What is [Field-pronoun] inherited retinal condition? - Selected Choice	1.00	6.00	2.03	1.47	2.16	246

#	Answer	%	Count
1	Retinitis Pigmentosa	42.68%	105
2	Stargardt Disease	44.72%	110
3	Achromatopsia	0.81%	2

4	Leber's Congenital Amaurosis	0.41%	1
5	Not sure	1.63%	4
6	Other	9.76%	24
	Total	100%	246

Q4.3 - Is [Field-pronoun] condition stationary (not changing significantly over time) or progressive (getting worse over time)?

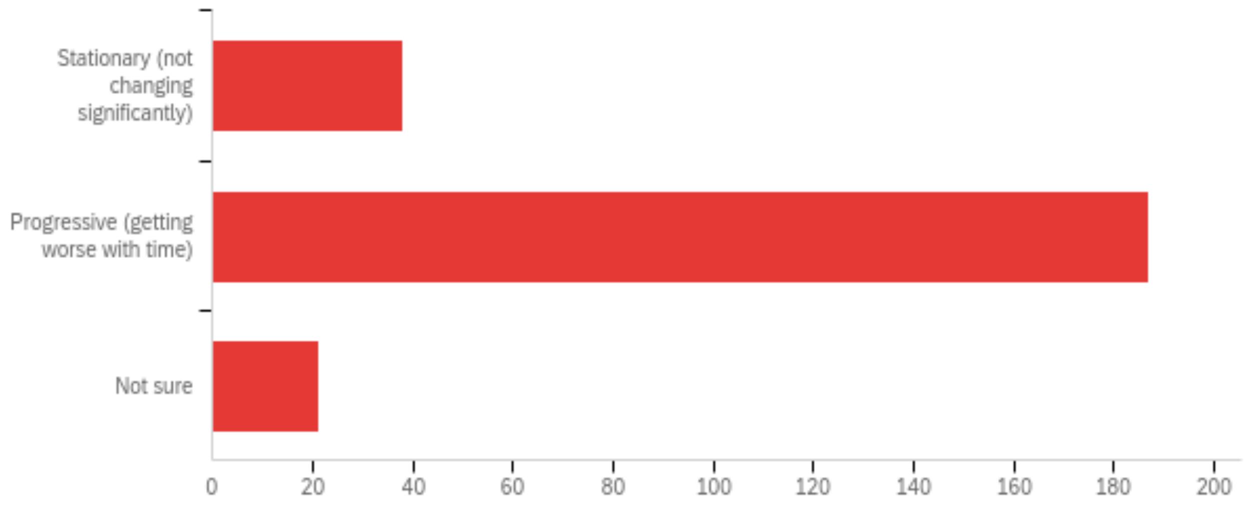

#	Field	Minimum	Maximum	Mean	Std Deviation	Variance	Count
1	Is [Field-pronoun] condition stationary (not changing significantly over time) or progressive (getting worse over time)?	1.00	3.00	1.93	0.48	0.24	246

#	Answer	%	Count
1	Stationary (not changing significantly)	15.45%	38
2	Progressive (getting worse with time)	76.02%	187
3	Not sure	8.54%	21
	Total	100%	246

Q4.4 - What gene is affected? The following list contains the 36 most commonly known genes in alphabetical order. "Other" and "Don't Know" are also options at the bottom of the list.

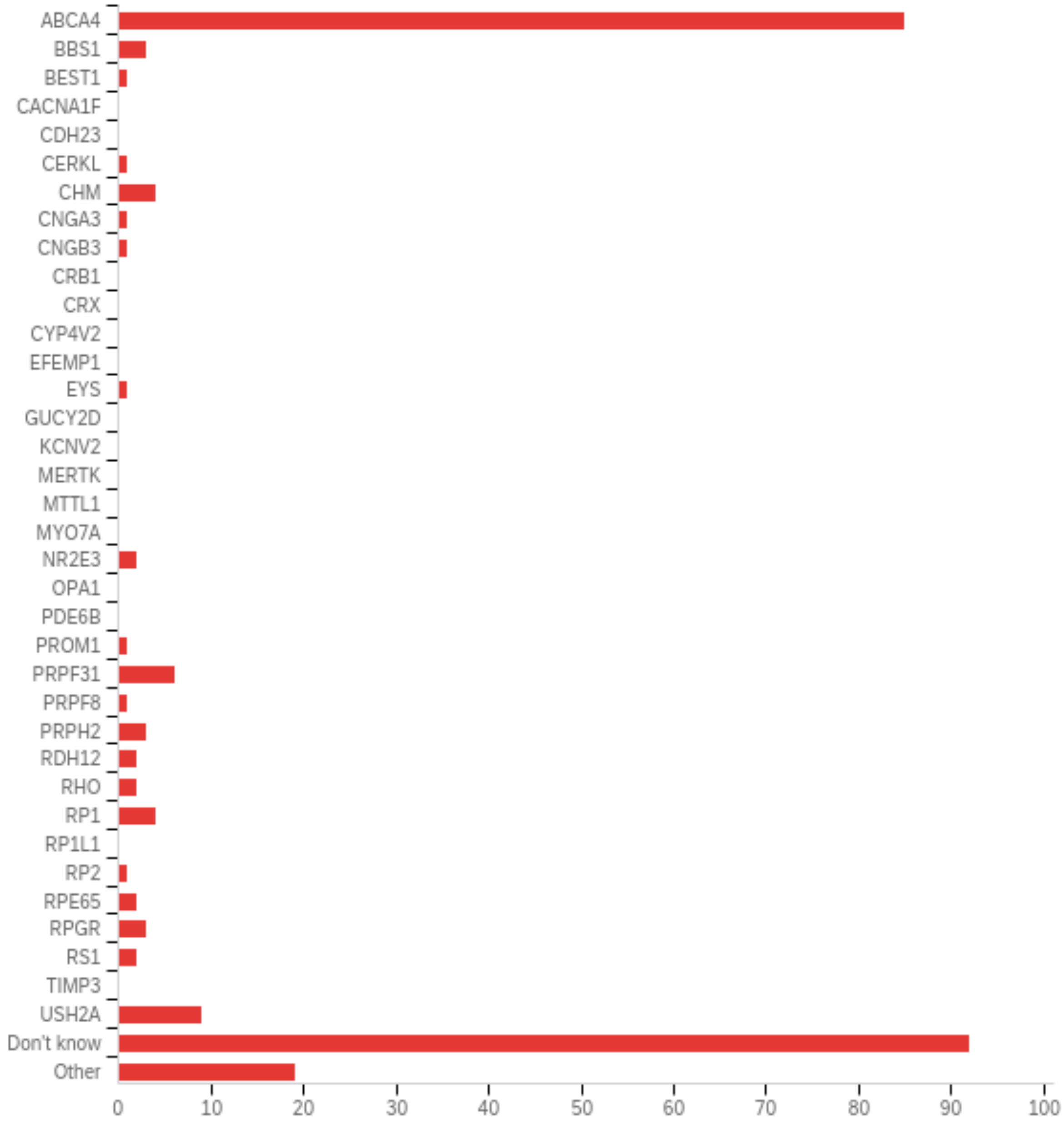

#	Field	Minimum	Maximum	Mean	Std Deviation	Variance	Count
1	Gene	1.00	38.00	21.98	16.78	281.70	246

#	Answer	%	Count
36	USH2A	3.66%	9
35	TIMP3	0.00%	0
34	RS1	0.81%	2
30	RP1L1	0.00%	0
33	RPGR	1.22%	3
32	RPE65	0.81%	2
31	RP2	0.41%	1
29	RP1	1.63%	4
28	RHO	0.81%	2
27	RDH12	0.81%	2
26	PRPH2	1.22%	3
24	PRPF31	2.44%	6
25	PRPF8	0.41%	1
23	PROM1	0.41%	1
22	PDE6B	0.00%	0
38	Other	7.72%	19
21	OPA1	0.00%	0
20	NR2E3	0.81%	2
19	MYO7A	0.00%	0
18	MTTL1	0.00%	0
17	MERTK	0.00%	0
16	KCNV2	0.00%	0
15	GUCY2D	0.00%	0
14	EYS	0.41%	1
13	EFEMP1	0.00%	0
37	Don't know	37.40%	92
12	CYP4V2	0.00%	0
11	CRX	0.00%	0
10	CRB1	0.00%	0

9	CNGB3	0.41%	1
8	CNGA3	0.41%	1
7	CHM	1.63%	4
6	CERKL	0.41%	1
5	CDH23	0.00%	0
4	CACNA1F	0.00%	0
3	BEST1	0.41%	1
2	BBS1	1.22%	3
1	ABCA4	34.55%	85
	Total	100%	246

Q4.7 - How do you describe [Field-pronoun] gender?

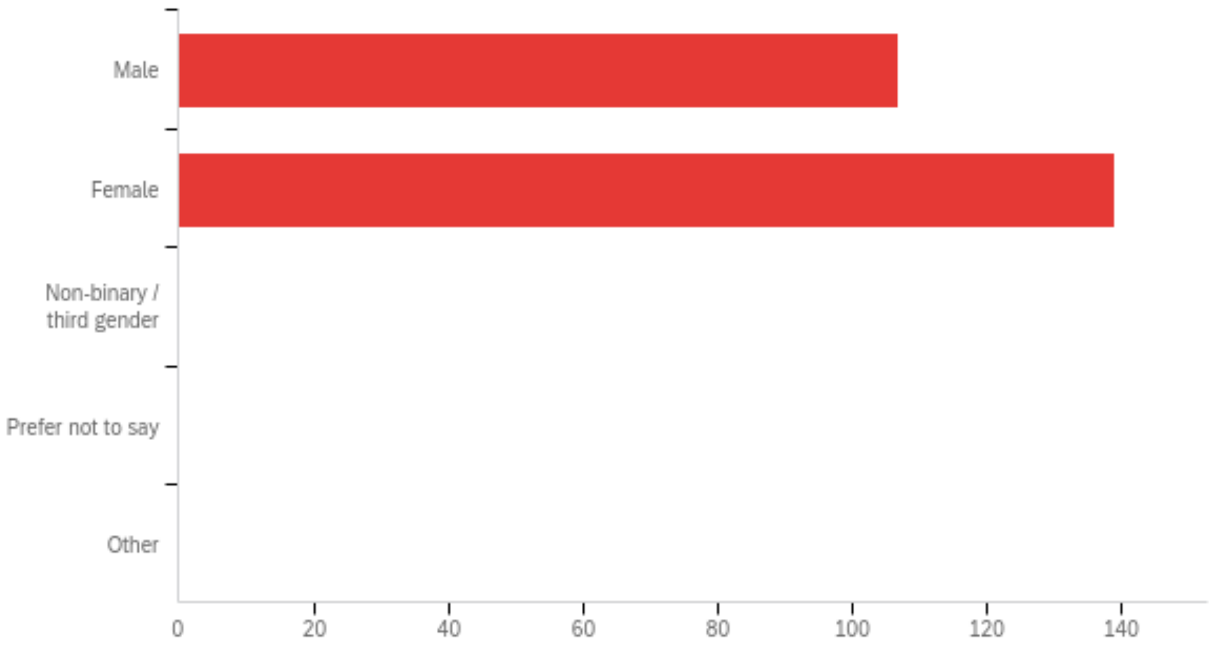

#	Field	Minimum	Maximum	Mean	Std Deviation	Variance	Count
1	How do you describe [Field-pronoun] gender? - Selected Choice	1.00	2.00	1.57	0.50	0.25	246

#	Answer	%	Count
1	Male	43.50%	107
2	Female	56.50%	139
3	Non-binary / third gender	0.00%	0
4	Prefer not to say	0.00%	0
5	Other	0.00%	0
	Total	100%	246

Q4.8 - What is [Field-pronoun] age? Please enter a number between 0 and 100.

#	Field	Minimum	Maximum	Mean	Std Deviation	Variance	Count
1	What is [Field-pronoun] age? Please enter a number between 0 and 100.	1.00	84.00	48.22	19.89	395.50	246

Q4.9 - What is [Field-pronoun] ethnicity?

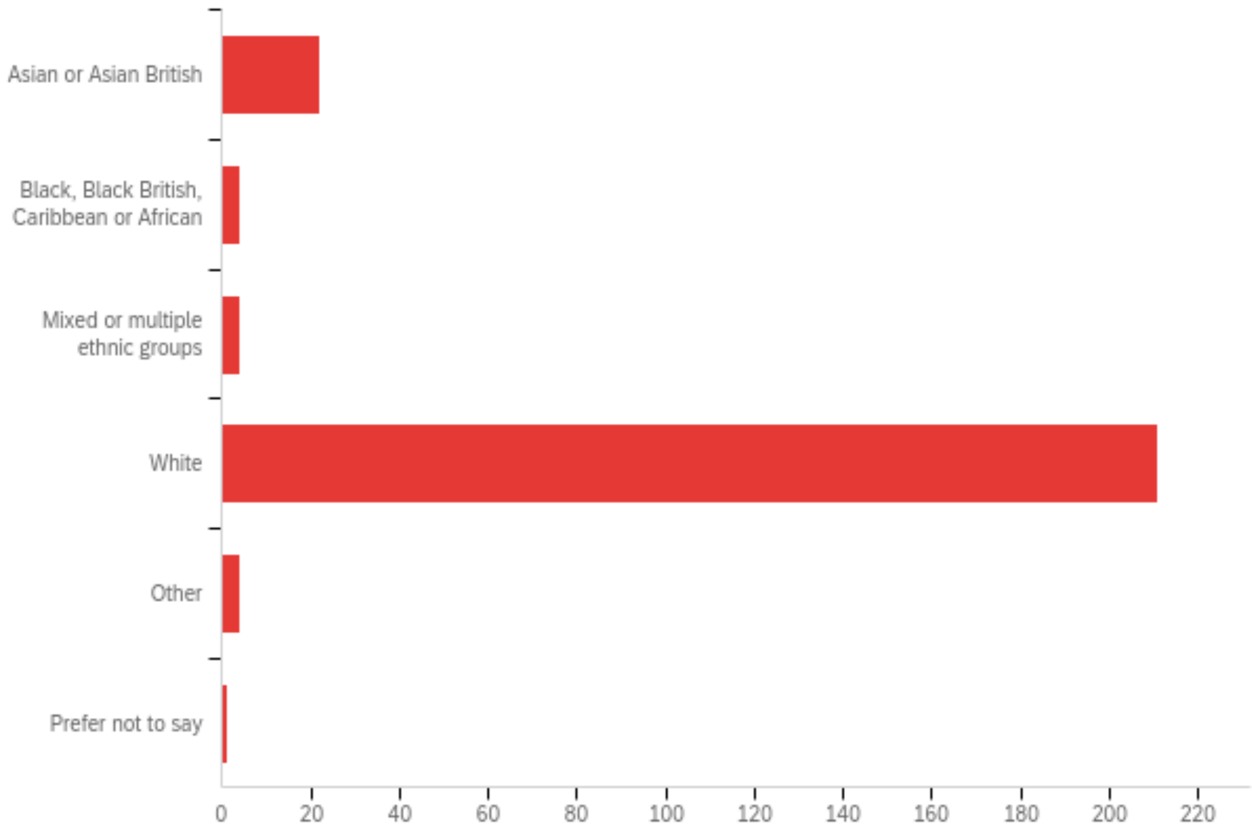

#	Field	Minimum	Maximum	Mean	Std Deviation	Variance	Count
1	What is [Field-pronoun] ethnicity? - Selected Choice	1.00	6.00	3.71	0.91	0.83	246

#	Answer	%	Count
1	Asian or Asian British	8.94%	22
2	Black, Black British, Caribbean or African	1.63%	4
3	Mixed or multiple ethnic groups	1.63%	4
4	White	85.77%	211
5	Other	1.63%	4
6	Prefer not to say	0.41%	1
	Total	100%	246

Q5.3 - How many years did it take [Field-pronoun3] to be seen by a specialist for inherited retinal diseases? Please try and guess as accurately as you can (number must be between 0 and 100.) Please round up to the closest year.

#	Field	Minimum	Maximum	Mean	Std Deviation	Variance	Count
1	How many years did it take [Field-pronoun3] to be seen by a specialist for inherited retinal diseases? Please try and guess as accurately as you can (number must be between 0 and 100.) Please round up to the closest year.	0.00	70.00	5.22	10.99	120.72	246

Q5.4 - How far did [Field-pronoun2] have to travel in miles from home to a specialist doctor? Please try and guess as accurately as you can.

#	Field	Minimum	Maximum	Mean	Std Deviation	Variance	Count
1	Miles	0.00	600.00	73.56	122.66	15045.78	242

Q5.2 - When were [Field-pronoun2] first diagnosed by an eye specialist? Please try and guess the year as accurately as you can (number must be between 1900 and 2024).

#	Field	Minimum	Maximum	Mean	Std Deviation	Variance	Count
1	When were [Field-pronoun2] first diagnosed by an eye specialist? Please try and guess the year as accurately as you can (number must be between 1900 and 2024).	1922.00	2024.00	2001.66	17.82	317.56	246

Q5.5 - Has [Field-pronoun] diagnosis changed since the first diagnosis?

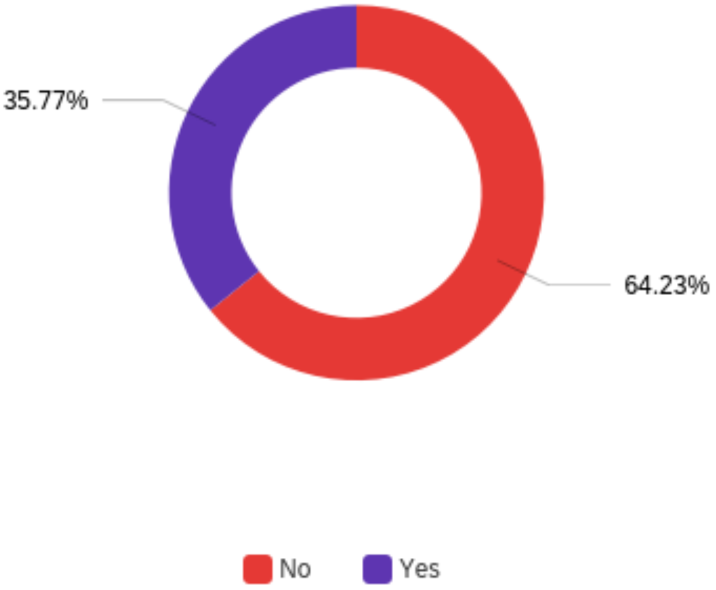

Q5.6 - How many hospitals or doctors (consultations) have [Field-pronoun2] seen in total to get a final diagnosis? Please enter the number as accurately as you can (number must be 1 or greater).

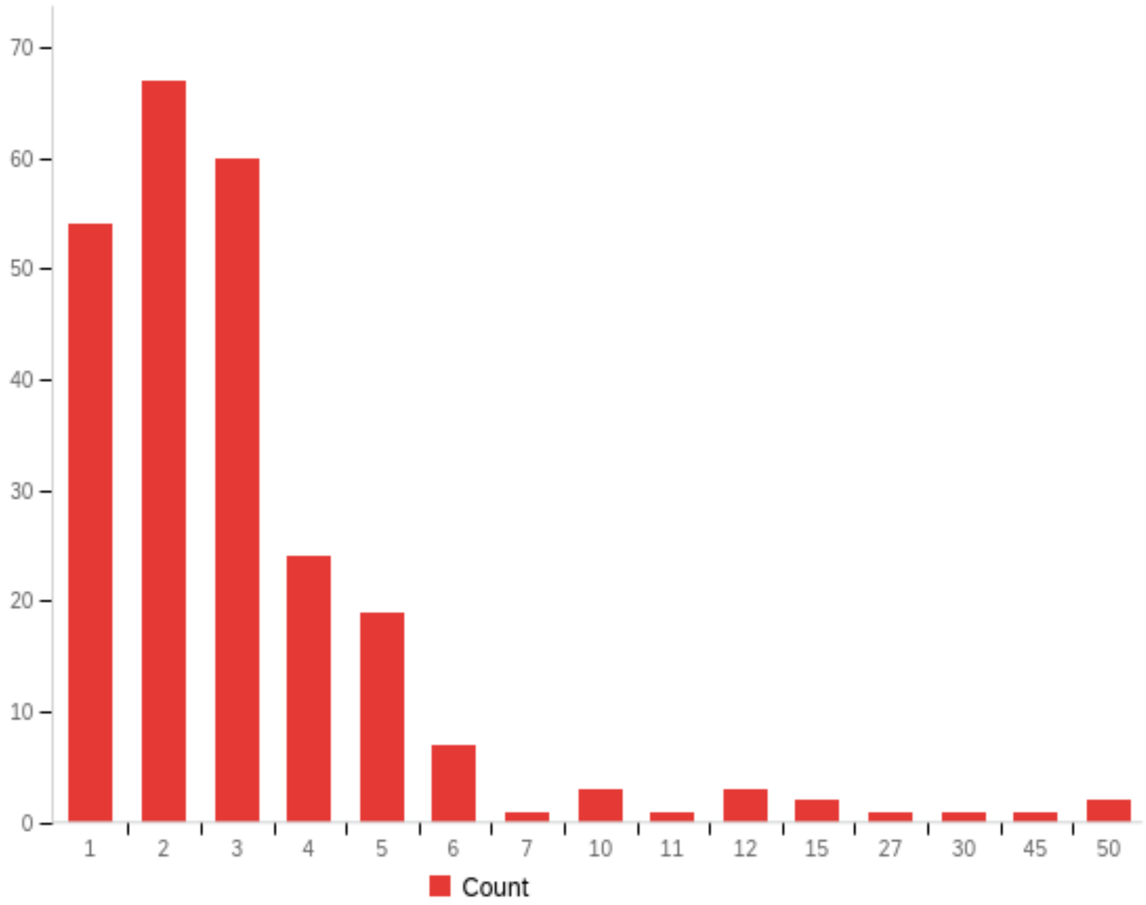

Q5.7 - Have [Field-pronoun2] been offered genetic testing?

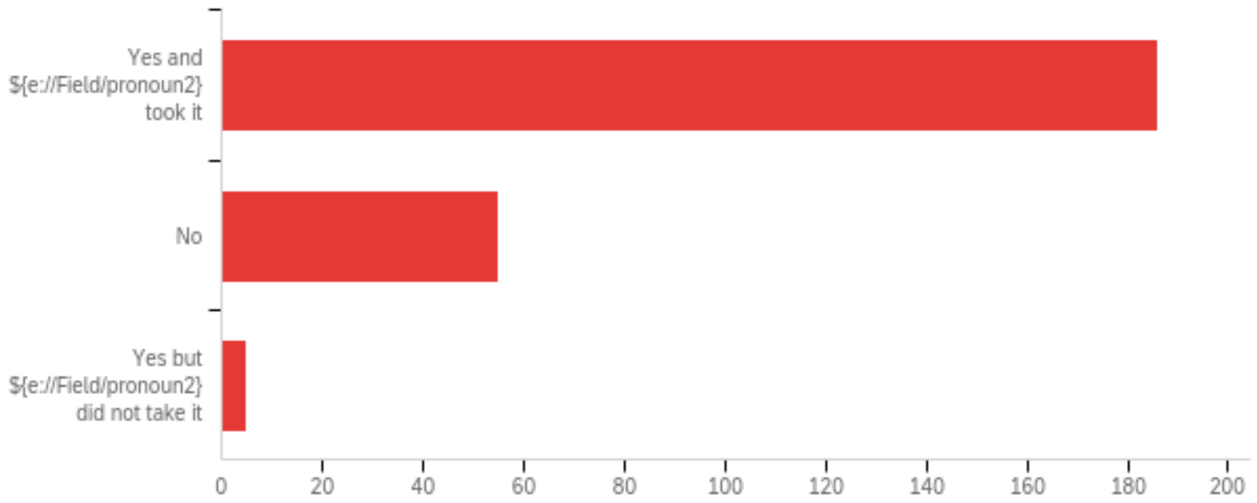

#	Field	Minimum	Maximum	Mean	Std Deviation	Variance	Count
1	Have [Field-pronoun2] been offered genetic testing? - Selected Choice	1.00	3.00	1.26	0.48	0.24	246

#	Answer	%	Count
1	Yes and ${e://Field/pronoun2} took it	75.61%	186
2	No	22.36%	55
3	Yes but ${e://Field/pronoun2} did not take it	2.03%	5
	Total	100%	246

Q5.13 - How were the genetic test results communicated to [Field-pronoun3]?

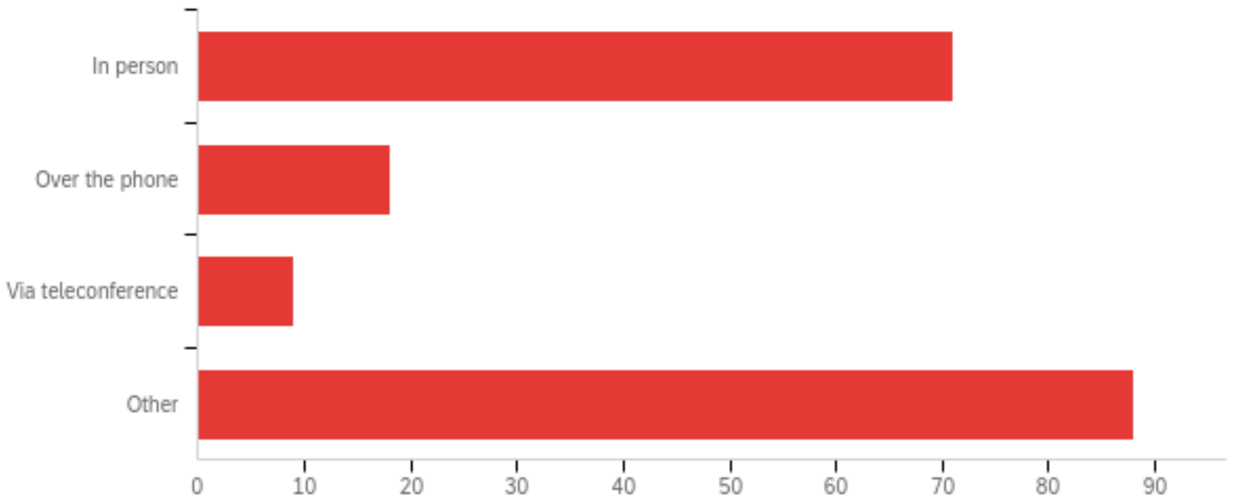

#	Field	Minimum	Maximum	Mean	Std Deviation	Variance	Count
1	How were the genetic test results communicated to [Field-pronoun3]? - Selected Choice	1.00	4.00	2.61	1.40	1.95	186

#	Answer	%	Count
1	In person	38.17%	71
2	Over the phone	9.68%	18
3	Via teleconference	4.84%	9
4	Other	47.31%	88
	Total	100%	186

Q5.14 - How long was the appointment in minutes where the genetic test results were communicated to [Field-pronoun3]? Please try to guess as accurately as you can. Please enter a number between 0 and 120 minutes (2 hours).

#	Field	Minimum	Maximum	Mean	Std Deviation	Variance	Count
1	How long was the appointment in minutes where the genetic test results were communicated to [Field-pronoun3]? Please try to guess as accurately as you can. Please enter a number between 0 and 120 minutes (2 hours).	0.00	120.00	25.15	24.88	618.96	175

Q5.15 - Do you feel like [Field-pronoun2] understand the genetic diagnosis?

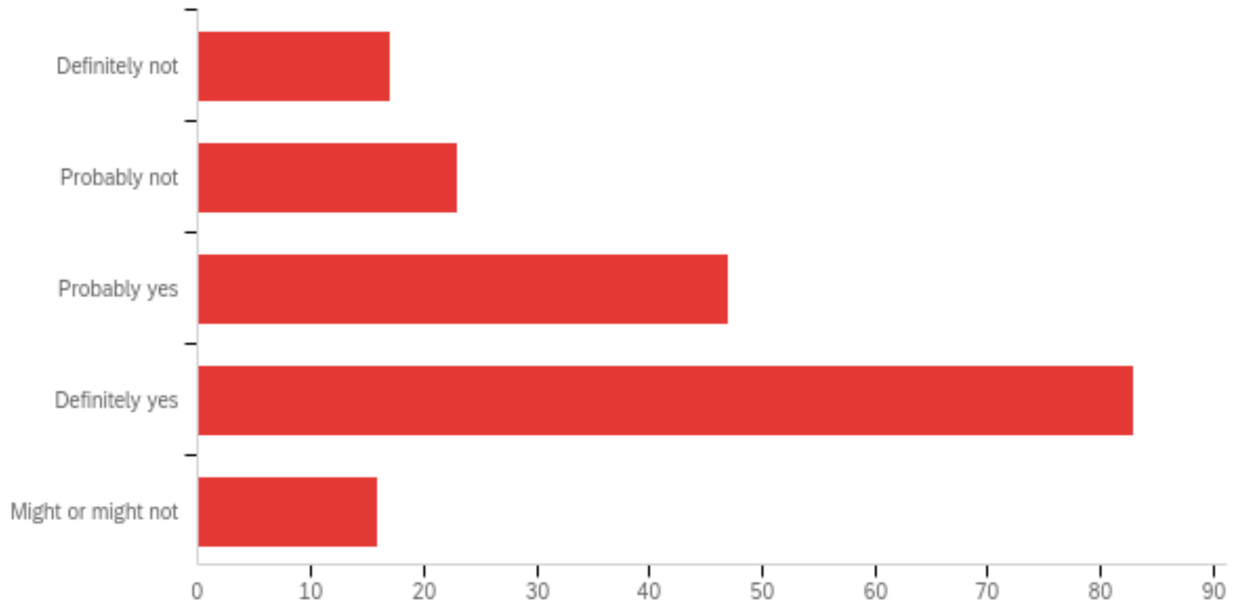

#	Field	Minimum	Maximum	Mean	Std Deviation	Variance	Count
1	Do you feel like [Field-pronoun2] understand the genetic diagnosis? - Selected Choice	1.00	7.00	4.18	1.58	2.50	186

#	Answer	%	Count
1	Definitely not	9.14%	17
2	Probably not	12.37%	23
4	Probably yes	25.27%	47
5	Definitely yes	44.62%	83
7	Might or might not	8.60%	16
	Total	100%	186

Q5.16 - Did you feel like [Field-pronoun] questions were answered?

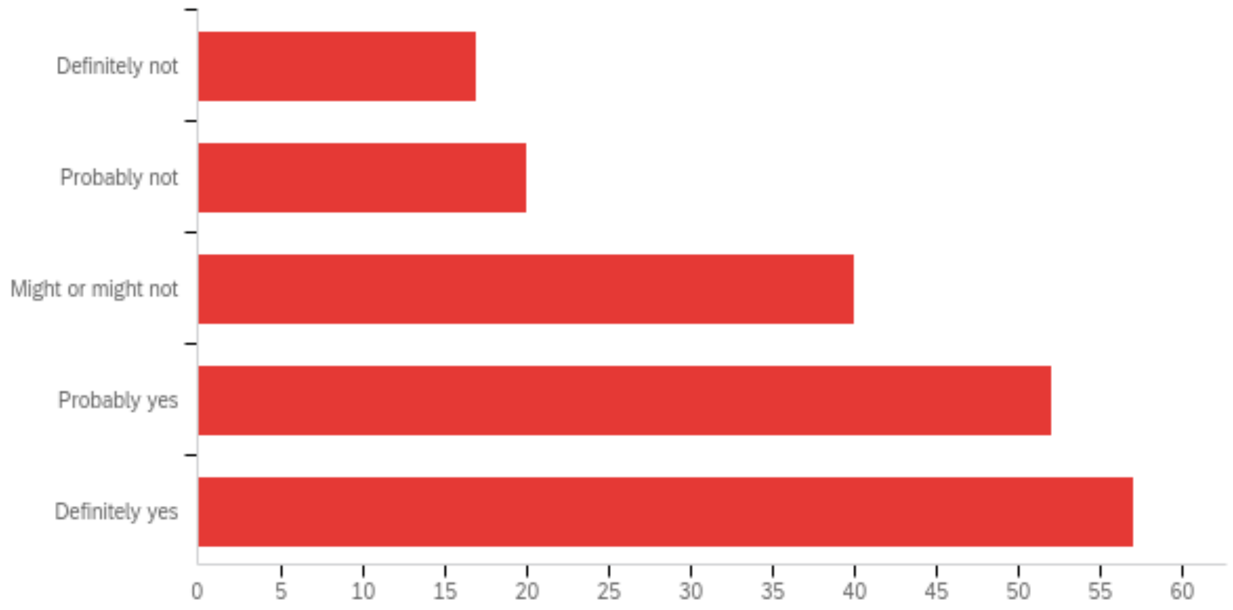

#	Field	Minimum	Maximum	Mean	Std Deviation	Variance	Count
1	Did you feel like [Field-pronoun] questions were answered? - Selected Choice	1.00	5.00	3.60	1.27	1.62	186

#	Answer	%	Count
1	Definitely not	9.14%	17
2	Probably not	10.75%	20
3	Might or might not	21.51%	40
4	Probably yes	27.96%	52
5	Definitely yes	30.65%	57
	Total	100%	186

Q6.1 - When attending a follow up visit, what sort of information do you think [Field-pronoun2] would like?

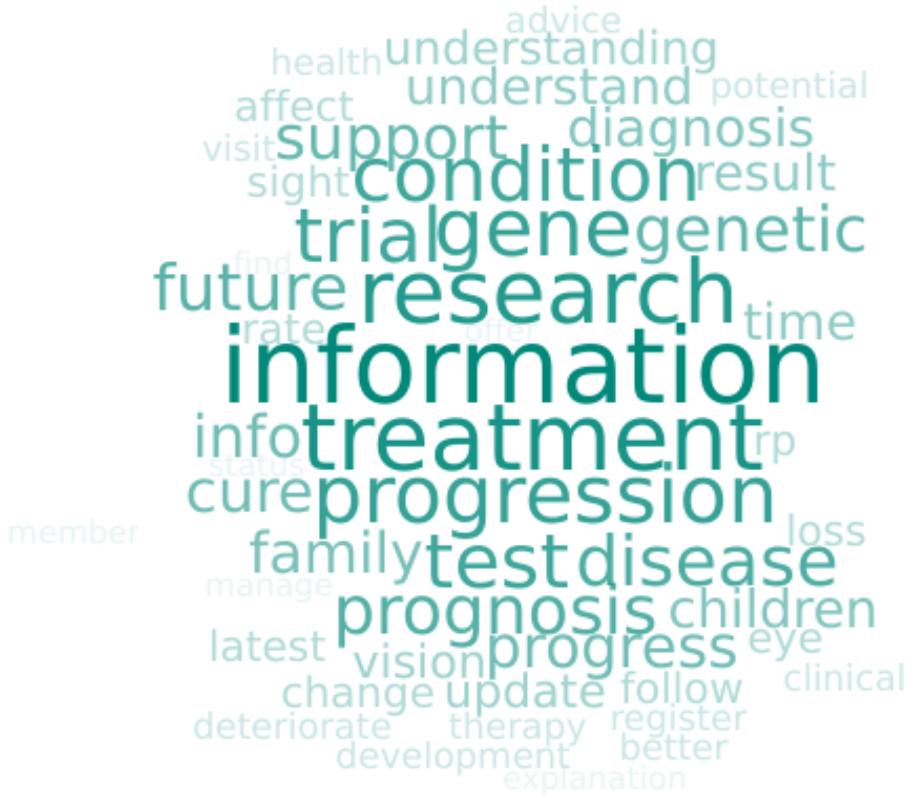

Q6.2 - Would [Field-pronoun2] like to also have a measure of progression as additional information rather than just having a doctor saying whether it's getting worse?

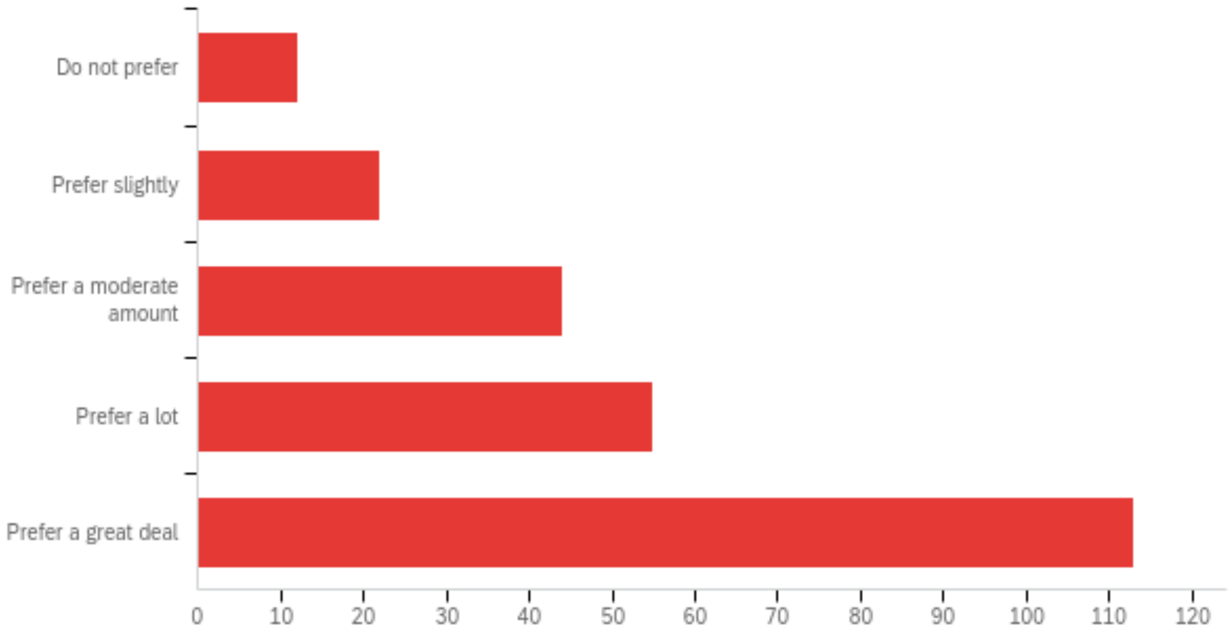

#	Field	Minimum	Maximum	Mean	Std Deviation	Variance	Count
1	Would [Field-pronoun2] like to also have a measure of progression as additional information rather than just having a doctor saying whether it's getting worse?	1.00	5.00	3.96	1.20	1.43	246

#	Answer	%	Count
1	Do not prefer	4.88%	12
2	Prefer slightly	8.94%	22
3	Prefer a moderate amount	17.89%	44
4	Prefer a lot	22.36%	55
5	Prefer a great deal	45.93%	113
	Total	100%	246

Q7.2 - How acceptable would it be for [Field-pronoun3] to get an eye scan-based assessment of whether [Field-pronoun2] might have an inherited retinal disease before a genetic test? Feel free to expand on your answer in the text box below.

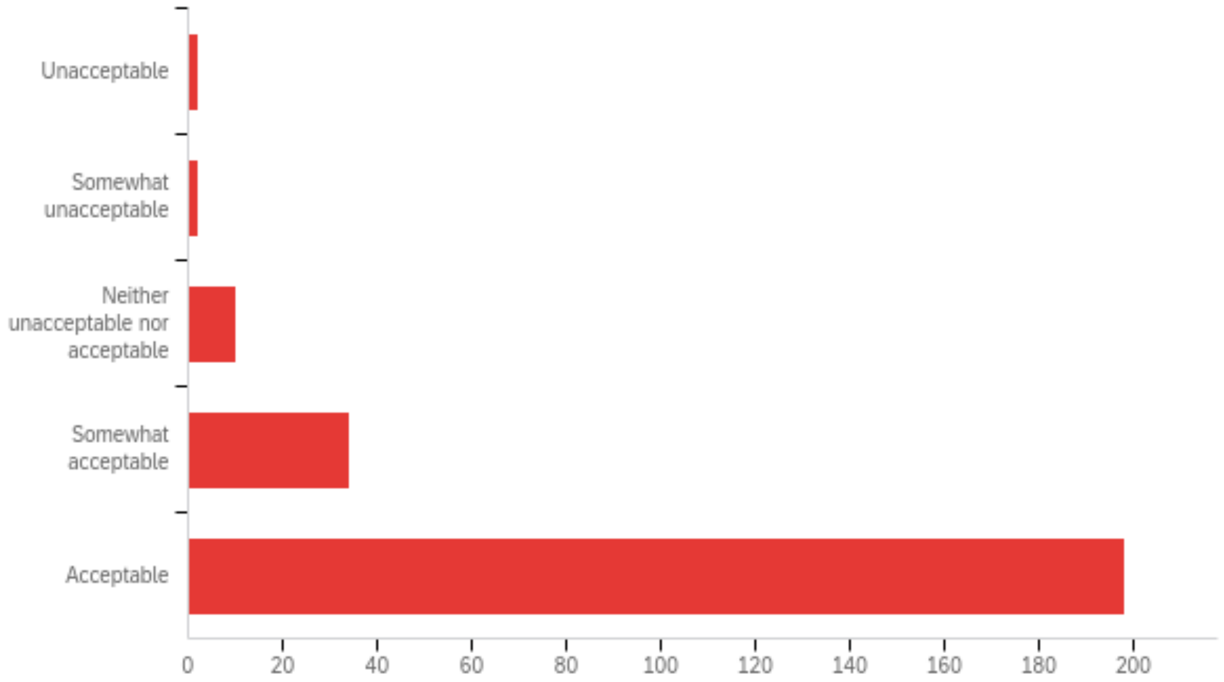

#	Field	Minimum	Maximum	Mean	Std Deviation	Variance	Count
1	How acceptable would it be for [Field-pronoun3] to get an eye scan-based assessment of whether [Field-pronoun2] might have an inherited retinal disease before a genetic test? Feel free to expand on your answer in the text box below. - Selected Choice	1.00	5.00	4.72	0.65	0.43	246

#	Answer	%	Count
1	Unacceptable	0.81%	2
2	Somewhat unacceptable	0.81%	2
3	Neither unacceptable nor acceptable	4.07%	10
4	Somewhat acceptable	13.82%	34
5	Acceptable	80.49%	198
	Total	100%	246

Q7.6 - How acceptable would it be for doctors to review [Field-pronoun] eye scans with the Eye2Gene AI?

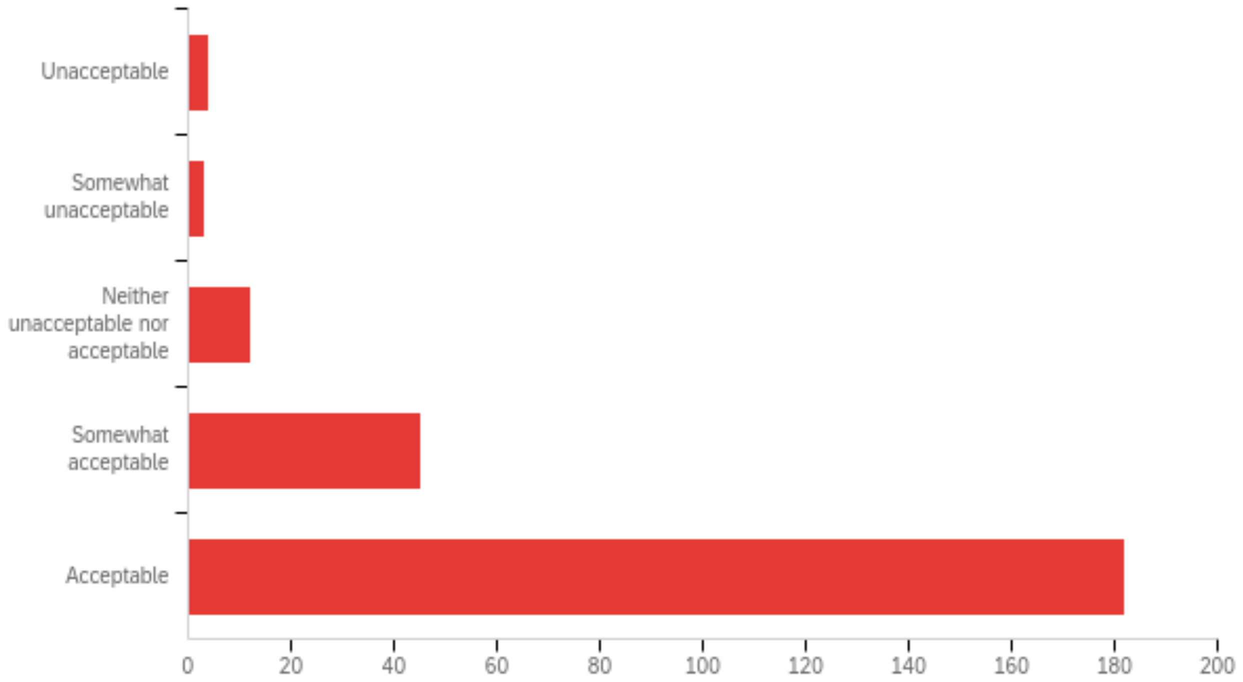

#	Field	Minimum	Maximum	Mean	Std Deviation	Variance	Count
1	How acceptable would it be for doctors to review [Field-pronoun] eye scans with the Eye2Gene AI?	1.00	5.00	4.62	0.78	0.60	246

#	Answer	%	Count
1	Unacceptable	1.63%	4
2	Somewhat unacceptable	1.22%	3
3	Neither unacceptable nor acceptable	4.88%	12
4	Somewhat acceptable	18.29%	45
5	Acceptable	73.98%	182
	Total	100%	246

Q7.3 - The Eye2Gene AI can help doctors identify the right diagnosis sooner. Do [Field-pronoun2] think this would be beneficial for [Field-pronoun3]? Feel free to expand on your answer in the text box below.

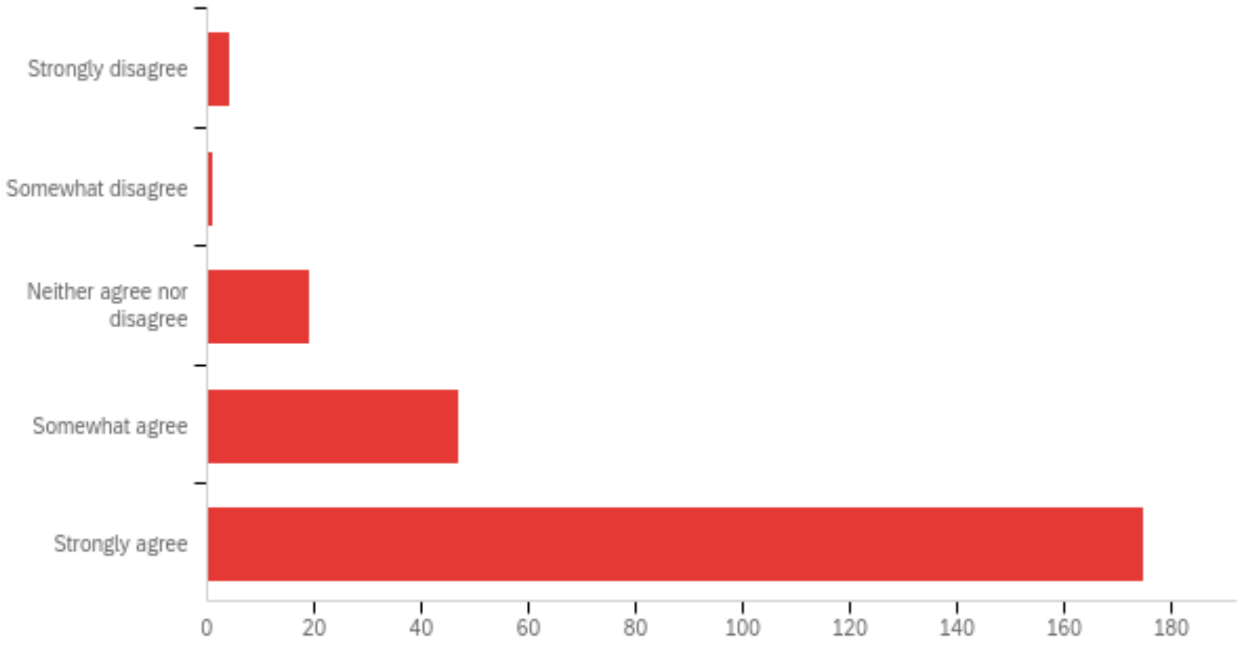

#	Field	Minimum	Maximum	Mean	Std Deviation	Variance	Count
1	The Eye2Gene AI can help doctors identify the right diagnosis sooner. Do [Field-pronoun2] think this would be beneficial for [Field-pronoun3]? Feel free to expand on your answer in the text box below. - Selected Choice	1.00	5.00	4.58	0.79	0.62	246

#	Answer	%	Count
1	Strongly disagree	1.63%	4
2	Somewhat disagree	0.41%	1
3	Neither agree nor disagree	7.72%	19
4	Somewhat agree	19.11%	47
5	Strongly agree	71.14%	175
	Total	100%	246

Q7.4 - Would [Field-pronoun2] like to have access to [Field-pronoun] retinal scans?

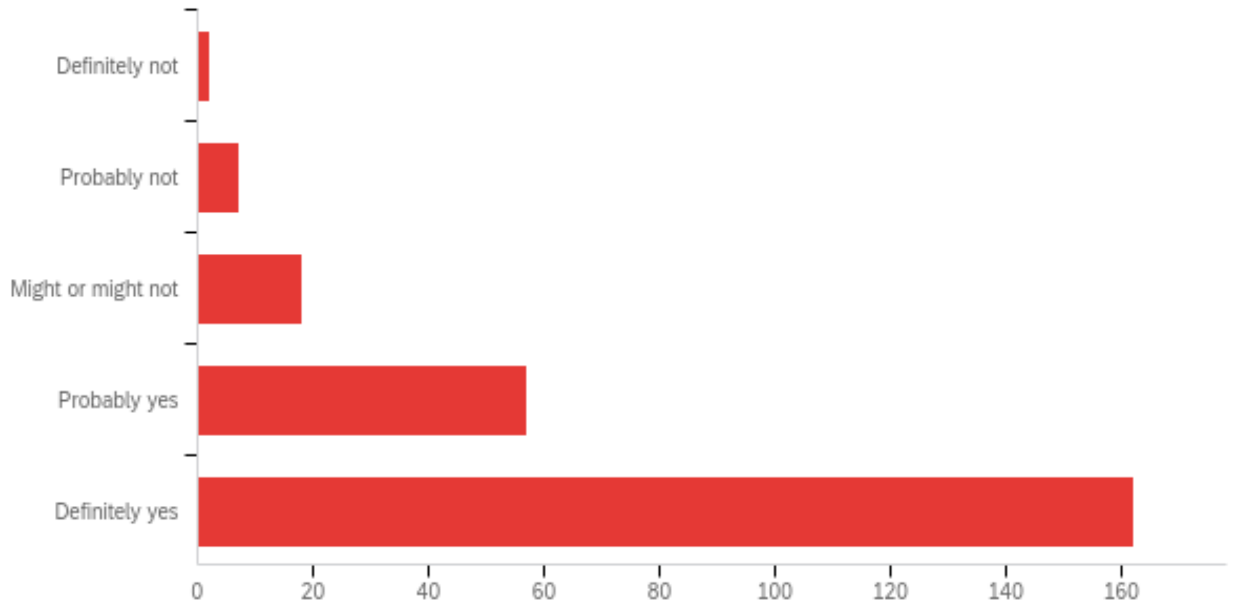

#	Field	Minimum	Maximum	Mean	Std Deviation	Variance	Count
1	Would [Field-pronoun2] like to have access to [Field-pronoun] retinal scans?	1.00	5.00	4.50	0.82	0.66	246

#	Answer	%	Count
1	Definitely not	0.81%	2
2	Probably not	2.85%	7
3	Might or might not	7.32%	18
4	Probably yes	23.17%	57
5	Definitely yes	65.85%	162
	Total	100%	246

Q7.5 - In order to avoid traveling and to save time, [Field-pronoun2] could have eye scans done closer to home. The eye scans would then be sent to the hospital for analysis. This would avoid having to commute to a hospital which might be far from [Field-pronoun] house. Is this something that [Field-pronoun2] would prefer?

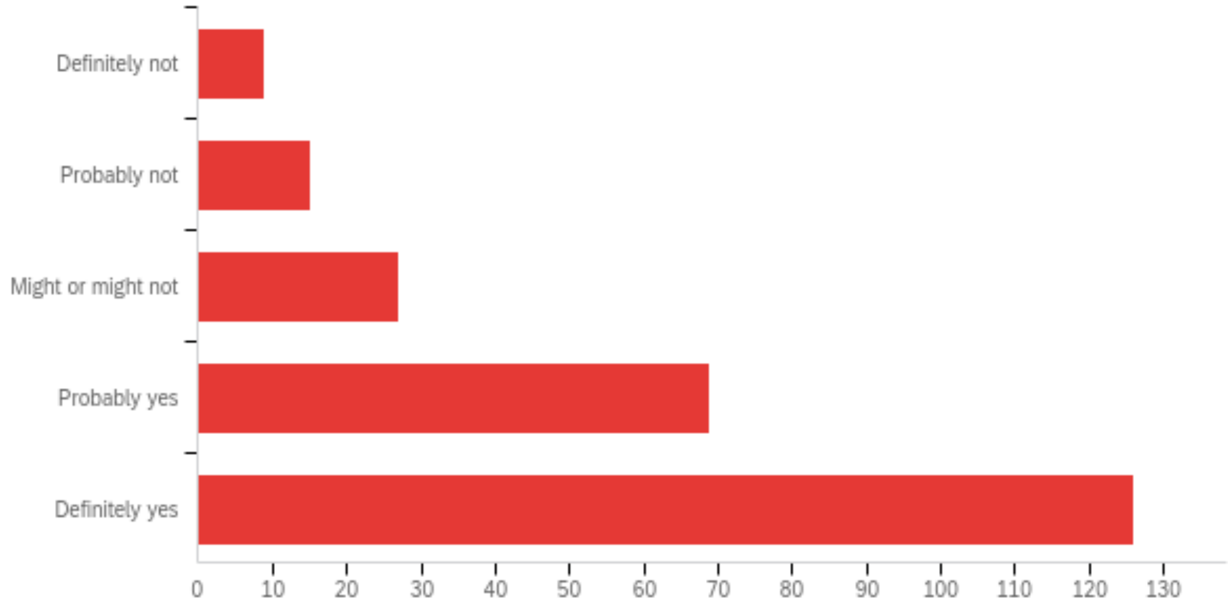

#	Field	Minimum	Maximum	Mean	Std Deviation	Variance	Count
1	In order to avoid traveling and to save time, [Field-pronoun2] could have eye scans done closer to home. The eye scans would then be sent to the hospital for analysis. This would avoid having to commute to a hospital which might be far from [Field-pronoun] house. Is this something that [Field-pronoun2] would prefer?	1.00	5.00	4.17	1.08	1.17	246

#	Answer	%	Count
1	Definitely not	3.66%	9
2	Probably not	6.10%	15
3	Might or might not	10.98%	27
4	Probably yes	28.05%	69
5	Definitely yes	51.22%	126
	Total	100%	246

Q5.9 - When did [Field-pronoun2] do a genetic test? Please enter year as accurately as you can (number must be between 1900 and 2024).

#	Field	Minimum	Maximum	Mean	Std Deviation	Variance	Count
1	When did [Field-pronoun2] do a genetic test? Please enter year as accurately as you can (number must be between 1900 and 2024).	1980.00	2024.00	2015.52	8.21	67.43	185

Q5.10 - In this section, we will ask you about the information that was provided to [Field-pronoun3] during/after the genetic testing process if the results of a genetic test have been received.

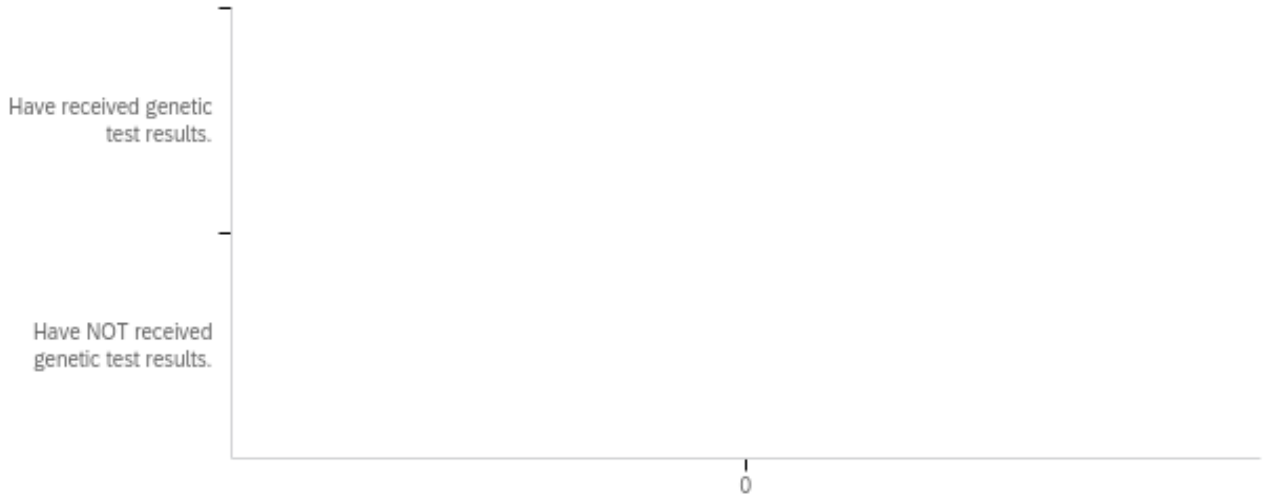

#	Field	Minimum	Maximum	Mean	Std Deviation	Variance	Count
1	In this section, we will ask you about the information that was provided to [Field-pronoun3] during/after the genetic testing process if the results of a genetic test have been received.	1.00	1.00	1.00	0.00	0.00	186

#	Answer	%	Count
2	Have received genetic test results.	0.00%	0
3	Have NOT received genetic test results.	0.00%	0
	Total		0

Q5.11 - When did [Field-pronoun2] receive genetic test results? Please enter year as accurately as you can (number must be between 1900 and 2024).

#	Field	Minimum	Maximum	Mean	Std Deviation	Variance	Count
1	When did [Field-pronoun2] receive genetic test results? Please enter year as accurately as you can (number must be between 1900 and 2024).	1900.00	2024.00	2015.27	13.91	193.61	186

Q5.12 - What was [Field-pronoun1] age when [Field-pronoun2] received their genetic test results? Please enter as accurately as you can.

#	Field	Minimum	Maximum	Mean	Std Deviation	Variance	Count
1	What was [Field-pronoun1] age when [Field-pronoun2] received their genetic test results? Please enter as accurately as you can.	1.00	73.00	38.59	19.30	372.57	184
